# The correlations of serum interleukin-6 (il-6) levels and serum soluble il-6 receptor levels with disease activity in systemic juvenile idiopathic arthritis patients with and without tocilizumab treatment

**DOI:** 10.1186/1546-0096-12-S1-P61

**Published:** 2014-09-17

**Authors:** Butsabong Lerkvaleekul, Sirisucha Soponkanaporn, Soamarat Vilaiyuk

**Affiliations:** 1 Pediatrics, Faculty of Medicine Ramathibodi Hospital, Mahidol University, Bangkok, Thailand

## Introduction

Interleukin-6 (IL-6) and soluble IL-6 receptor (sIL-6R) have been demonstrated to play a significant role as inflammatory mediators in systemic juvenile idiopathic arthritis (SJIA). Tocilizumab, a humanized anti-IL-6 receptor antibody, becomes a new biologic treatment for SJIA nowadays but the correlation of serum IL-6 levels and serum sIL-6R levels with disease activity in SJIA with and without tocilizumab treatment are still unclear.

## Objectives

To determine the correlations of serum IL-6 levels and serum sIL-6R levels with disease activity in SJIA patients with and without tocilizumab treatment

## Methods

SJIA patients in pediatric rheumatology clinic, Ramathibodi hospital between September 2011 and June 2013 were enrolled in this study. Patients were followed up three times in 2-3 months interval. Fifteen healthy children were included as normal controls. Demographic data was collected. During the visit, patients were evaluated according to Juvenile Arthritis Disease Activity Score-71 (JADAS-71) and blood samplings were collected for complete blood count, erythrocyte sedimentation rate, IL-6 levels, and sIL-6R levels, then patients were categorized into 4 groups; 1) active disease with systemic features and arthritis 2) active disease with only arthritis 3) remission on medication 4) remission off medication

## Results

Forty-two SJIA patients, 131 blood samplings were included in this study. Seventeen patients (40%) were treated with tocilizumab during the study. Serum IL-6 levels in patients without tocilizumab treatment significantly elevated in active disease with systemic features and arthritis [median (IQR) = 101.8 (303.2) pg/mL] when compared to active disease with only arthritis [median (IQR) = 4.5 (23) pg/mL], and remission on medication [median (IQR) = 1.5 (0.55) pg/mL], whereas serum IL-6 levels in patients with tocilizumab treatment were not different between groups but there were significantly different when compared to healthy children (p < 0.05). In addition, the correlation between serum IL-6 levels and JADAS-71 in patients without tocilizumab treatment (r = 0.71, p < 0.001) was stronger than patients with tocilizumab treatment (r = 0.42, p = 0.01). Serum sIL-6R levels in SJIA patients with and without tocilizumab treatment were significantly higher when compared to healthy children (p < 0.05). Interestingly, in patients with tocilizumab treatment, serum sIL-6R levels were extremely higher [median (IQR) = 1,110.3 (840.2) ng/mL] than patients without tocilizumab treatment [median (IQR) = 94.2 (82.7) ng/mL].

## Conclusion

The correlation between serum IL-6 levels and disease activity in patients without tocilizumab treatment was stronger than patients with tocilizumab treatment. In addition, serum sIL-6R levels in patients with tocilizumab treatment were extremely higher than patients without tocilizumab treatment.

## Disclosure of interest

None declared.

